# Clinical and Serological Characteristics of Idiopathic Inflammatory Myopathies According to the Presence of Interstitial Lung Disease and Initial Evaluating Medical Specialty: A Single-Center Experience

**DOI:** 10.3390/jpm16060311

**Published:** 2026-06-08

**Authors:** Christina Koukouvitaki, Sofia Flouda, Theofanis Karageorgas, Stelios Loukides, Dimitrios T. Boumpas, Antonis Fanouriakis, Aggelos Banos, Vasilios Tzilas

**Affiliations:** 1 Rheumatology Unit, University Hospital “Attikon”, Athens Medical School, National and Kapodistrian University of Athens, 10431 Athens, Greece; christinakoukouvitaki1@gmail.com (C.K.); sofieflou1991@gmail.com (S.F.); tkarageorgas@gmail.com (T.K.); boumpasd@med.uoa.gr (D.T.B.); afanour@med.uoa.gr (A.F.); abanos@med.uoa.gr (A.B.); 2 2nd Pulmonary Medicine Department, University Hospital “Attikon”, Athens Medical School, National and Kapodistrian University of Athens, 10431 Athens, Greece; loukstel@med.uoa.gr; 3 Laboratory of Autoimmunity and Inflammation, Center for Clinical, Biomedical Research Foundation, Experimental Surgery and Translational Research, Academy of Athens, 10431 Athens, Greece

**Keywords:** idiopathic inflammatory myopathies, interstitial lung disease, antisynthetase syndrome, myositis-specific autoantibodies, clinically amyopathic myositis, personalized medicine

## Abstract

**Background**: Idiopathic inflammatory myopathies (IIMs) are a heterogenous group of disorders frequently complicated by interstitial lung disease (ILD). We sought to discern phenotypic and serological differences according to the presence of ILD and initial evaluating medical specialty, i.e., rheumatology vs. pulmonology, with the goal of advancing personalized medicine. **Methods**: A computer-assisted search was conducted to identify patients with a diagnosis of IIM seen at Attikon University Hospital, from January 2010 to December 2025. Medical records were reviewed for clinical, laboratory and serological features. **Results**: We identified 140 patients with IIM; 96 (68.6%) were female with a mean age at diagnosis of 55.8 years (SD 15.7). ILD was present in 75 patients (53.6%), being more common among males (30/44, 68.2% vs. 45/96 females, 46.9%, *p* = 0.019). Patients in the ILD subgroup were older at diagnosis (mean age 60.2 years vs. 50.7 years, *p* < 0.001) and presented more often with dyspnea (41 vs. 1, *p* < 0.001), higher CRP (median 5.95 mg/L vs. 2.9 mg/L, *p* = 0.024), and lower CPK (median 103 vs. 580, *p* < 0.001). Patients first seen by a pulmonologist were more likely to be older (mean age 60.5 years vs. 53 years, *p* = 0.002) and to present with dyspnea (33 vs. 9, *p* < 0.001) and ILD (48 vs. 27, *p* < 0.001). By contrast, skin involvement (61% vs. 27%, *p* = 0.04), muscle weakness (53 vs. 15, *p* < 0.001) and elevated CPK (median 301.5 vs. 103.5, *p* = 0.013) were less frequent in these patients as compared to patients first evaluated by a rheumatologist. Anti-tRNA synthetase, anti-Ro52 and anti-Pm/Scl antibodies were more frequent in the ILD subgroup. Anti-tRNA antibodies were also more frequent in patients first seen by a pulmonologist. **Conclusions**: Patients with IIM-ILD are more likely to present without overt clinical or biochemical characteristics of muscle involvement, thereby increasing the likelihood of initial evaluation by pulmonologists.

## 1. Introduction

Idiopathic inflammatory myopathies (IIMs) are a heterogeneous group of systemic autoimmune disorders characterized by multi-organ involvement, primarily affecting skeletal muscle, but also involving the skin, lungs, joints, heart, and gastrointestinal tract [1,2]. Interstitial lung disease (ILD) is one of the most significant extramuscular manifestations and a major contributor to morbidity and mortality in affected patients [3,4].

The prevalence of ILD in patients with IIMs varies widely across studies, occurring in 20–80% [5,6] depending on the disease subtype, autoantibody profile, and diagnostic methods. In the context of antisynthetase syndrome (ASyS), ILD is a cardinal manifestation, occurring in more than 80% of patients [7], and is associated with worse functional status, increased hospitalization, and reduced survival, particularly in patients with rapidly progressive ILD [3,6,8]. Importantly, ILD can occasionally precede the onset of muscle symptoms, often leading to diagnostic delays or referral to different medical specialties [1]. As a result, patients may initially present to either pulmonologists or rheumatologists, potentially influencing diagnostic pathways and early management strategies.

While several studies have examined ILD in IIMs, data exploring the relationship between ILD status, initial specialist referral, and patient characteristics remain limited. A better understanding of these differences may provide insight into disease phenotypes, causes for diagnostic delay, and factors affecting patient outcomes. With the aim of improving the concept of personalized medicine, the present study compares patients with IIMs according to the presence or absence of ILD and the kind of medical specialty that first assessed the patient at symptom onset, focusing on demographic, clinical, and laboratory characteristics.

## 2. Materials and Methods

### 2.1. Patient Population and Data Collection

A computer-assisted search was conducted to identify patients with a diagnosis of IIM encountered at Attikon University Hospital, from January 2010 to December 2025. The study was approved by the Institutional Ethics Committee of Attikon University Hospital (protocol number 103/06-03-2014).

Medical records were retrospectively reviewed to identify clinical, laboratory and serological parameters at the time of IIM diagnosis. The diagnosis of IIM was based on the 2017 European League Against Rheumatism/American College of Rheumatology (EULAR/ACR) classification criteria for adult and juvenile idiopathic inflammatory myopathies and their major subgroups [9]. ASyS was diagnosed according to Connors’ criteria [10], while only patients in whom ILD was diagnosed based on HRCT findings were included [11].

We collected demographic and clinical data including age at diagnosis, sex, smoking status, respiratory and systemic symptoms, physical findings, and hematologic and serological parameters. The patients were classified into subgroups according to (i) the presence or absence of ILD and (ii) the initial evaluation medical specialty (rheumatologist versus pulmonologist). Antibody profiles were obtained using the EUROLINE Autoimmune Inflammatory Myopathies 16 Ag [IgG] test kit, which provides a qualitative assay for human autoantibodies of the immunoglobulin class IgG to 16 different antigens in serum or plasma: Mi-2α, Mi-2β, TIF1γ, MDA5, NXP2, SAE1, Ku, PM-Scl100, PM-Scl75, Jo-1, SRP, PL-7, PL-12, EJ, OJ, and Ro-52.

### 2.2. Statistical Analysis

Categorical variables are presented as numbers (percentages), with the latter calculated based on available data and within each subgroup. For sex and smoking status comparisons, percentages were calculated within sex and smoking categories, due to unequal group sizes. Normality of continuous variables was assessed using the Shapiro–Wilk test and visual inspection of Q–Q plots. Continuous variables with normal distributions are presented as the mean ± standard deviation (SD), whereas non-normally distributed variables are presented as the median and interquartile range (IQR).

Categorical variables were compared using the chi-square test or Fisher’s exact test, as appropriate. Comparisons between two independent groups of continuous variables were performed using the independent-samples *t*-test for normally distributed variables and the Mann–Whitney U test for non-normally distributed variables. Equality of variances was assessed using Levene’s test. No formal adjustment for multiple testing was applied, as the nature of the analysis was exploratory. A *p*-value < 0.05 was considered statistically significant for all comparisons. Due to skewed distributions, laboratory values were log-transformed (natural logarithm, ln) for graphical presentation. All data were recorded and analyzed using IBM SPSS Statistics software, version 29.0.2.0.

## 3. Results

### 3.1. General Cohort Characteristics

A total of 140 patients were included; 44 (31.4%) were males and 96 (68.6%) females (Supplementary Figure S1A). The mean ± SD age at diagnosis was 55.8 ± 15.7 years. Most patients were never smokers (69/119, 58%), whereas 20 (16.8%) were current and 30 (25.2%) former smokers (Supplementary Figure S1B). At initial presentation, 88 (62.9%) patients had skin manifestations, 74 (52.9%) arthritis, 69 (49.3%) muscle weakness, 42 (30%) dyspnea, and 28 (20%) Raynaud’s phenomenon (Supplementary Figure S1C). Baseline laboratory values are summarized in Table 1 and Supplementary Figure S1D. Anti-tRNA was detected in 42 patients (30%), anti-MDA5 in 15 (10.7%), anti-SRP in 10 (7.1%), anti-SAE1 in four (2.9%), anti-Mi-2 in 11 (7.9%), anti-NXP-2 in nine (6.9%), anti-Tiff1-gamma in seven (5.0%), anti-HMGCR in four (2.9%), anti-cN1a in eight (5.7%), anti-Ro52 in 43 (30.7%), anti-PM/Scl in 14 (10.0%), and anti-Ku in seven (5.0%) (Supplementary Figure S1E). The frequency of myositis-specific and myositis-associated autoantibodies is also shown in Table 1.

### 3.2. Dyspnea and Anti-tRNA Synthetase and Anti-Ro52 Positivity Characterize ILD Subgroup of Patients with IIM

**Figure 1:**
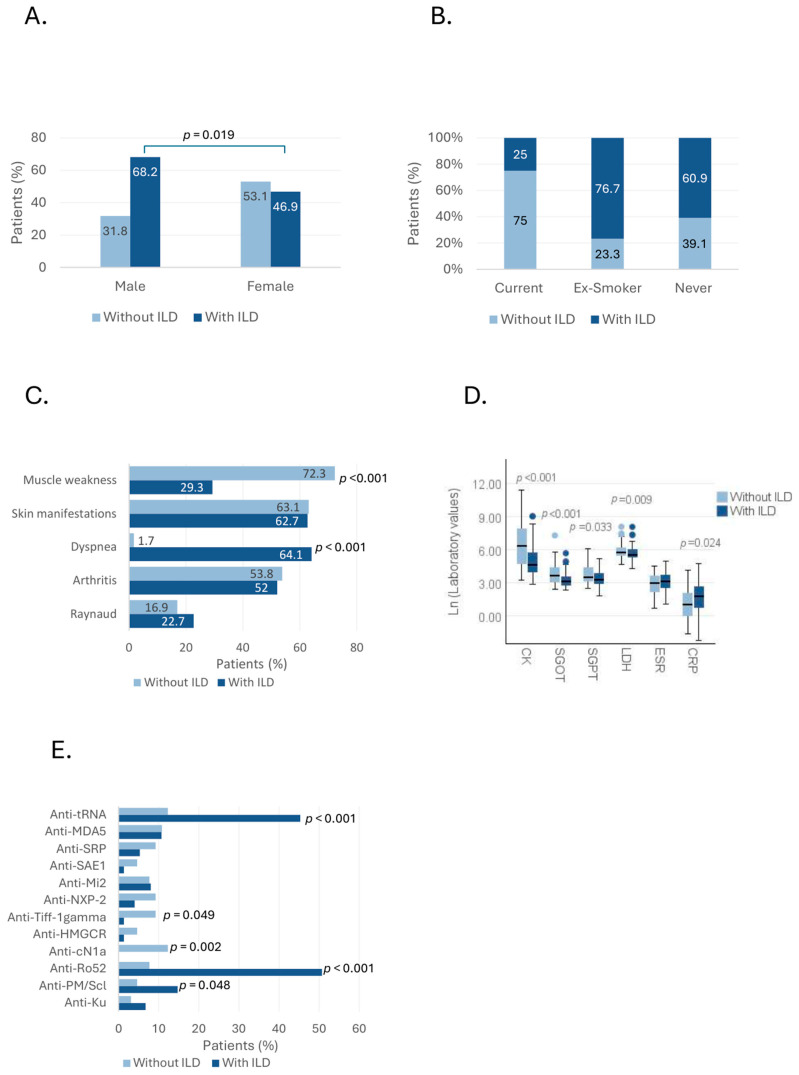
Demographic, clinical and serological characteristics of ILD and non-ILD subgroups of patients with IIM. Bar and Box plots depict differences between ILD and non-ILD subgroups in terms of (**A**) sex distribution, (**B**) smoking, (**C**) symptoms, (**D**) laboratory findings and (**E**) auto-antibody profile. For laboratory findings, boxes represent the interquartile range (IQR), with the horizontal line indicating the median. Whiskers extend to 1.5×/QR, and circles represent outliers. Values are displayed on the natural logarithmic (In) scale for visualization. Statistical analyses were performed on the original data. *p*-values were calculated using the x^2^ test or Fisher’s exact test, as appropriate. For laboratory findings, *p*-values were calculated using the Mann–Whitney U test.

In our cohort, 53.6% (75/140) of patients had ILD. In comparisons of the ILD vs. non-ILD subgroups, patients with ILD were older at diagnosis [mean (SD): 60.2 (12.5) vs. 50.7 (17.4) years for those without ILD, *p* < 0.001], while ILD was more frequent among men with IIM [30/44 (68.2%) had ILD vs. 45/96 women (46.9%), OR = 2.43, 95% CI: 1.15–5.15, *p* = 0.019] (Figure 1A).

Smoking status differed significantly between the groups (valid n = 119, missing n = 21). In the ILD subgroup, five (25%) were current smokers, 23 (76.7%) former smokers, and 42 (60.9%) never smokers (*p* = 0.001 compared to the non-ILD subgroup) (Figure 1B).

Dyspnea was more frequently the initial manifestation in patients with ILD [41 (64.1%) vs. 1 (1.7%), *p* < 0.001], whereas muscle weakness was less common in this subgroup [22 (29.3%) vs. 47 (72.3%), *p* < 0.001] (Figure 1C). No significant differences were observed for skin manifestations, arthritis, or Raynaud’s phenomenon.

Creatine kinase (CK) levels were lower in the ILD subgroup [median 103 IU/L (IQR 50.8–331.8) vs. 580 IU/L (IQR 107.5–2674.5), *p* < 0.001], as were SGOT, SGPT and LDH levels (Table 1, Figure 1D). In contrast, CRP was higher in these patients [median 5.95 mg/L (IQR 2.0–14.1) vs. 2.9 mg/L (IQR 1.0–8.0), *p* = 0.024].

Regarding autoantibodies, anti-tRNA synthetase, anti-Ro52 and anti-Pm/Scl positivity were more frequent in the ILD subgroup, while anti-Tiff1-gamma positivity was more frequent in the non-ILD subgroup. All eight patients positive for anti-cN1A belonged to the non-ILD subgroup (*p* = 0.002) (Figure 1E). Demographic, clinical and serological characteristics according to ILD status are shown in Table 1.

### 3.3. Medical Specialty of First Assessment Is Associated with Different Clinical Manifestations and Serological Profile in Patients with IIM

**Figure 2:**
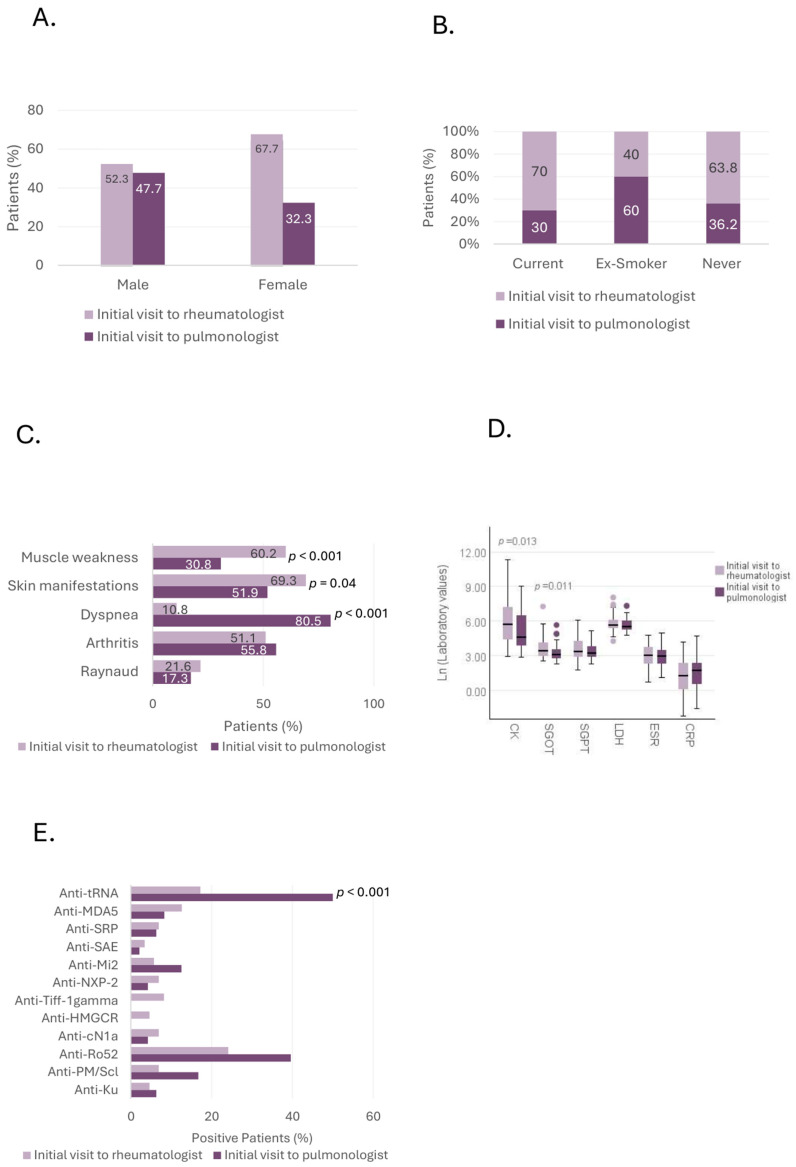
Comparisons of demographic, clinical and serological characteristics of patients with IIM, based on the specialty of first evaluation. Bar and Box plots depict differences between the ILD and non-ILD subgroups in terms of (**A**) sex distribution, (**B**) smoking, (**C**) symptoms, (**D**) laboratory findings and (**E**) auto-antibody profile. For laboratory findings, boxes represent the interquartile range (IQR), with the horizontal line indicating the median. Whiskers extend to 1.5×/QR, and circles represent outliers. Values are displayed on the natural logarithmic (In) scale for visualization. Statistical analyses were performed on the original data. *p*-values were calculated using the x^2^ test or Fisher’s exact test, as appropriate. For laboratory findings, *p*-values were calculated using the Mann–Whitney U test.

Patients first evaluated by a pulmonologist were older [mean (SD): 60.5 (11.2) vs. 53 (17.3) years, *p* = 0.002], while there were no significant sex differences between the subgroups [21/44 males (47.7%) vs. 31/96 females (32.3%) were first evaluated by a pulmonologist, *p* = 0.079] (Figure 2A). Smoking status differed between the groups. In the pulmonology subgroup, six patients (30%) were current smokers, 18 (60%) former smokers, and 25 (36.2%) never smokers (*p* = 0.047 compared to the rheumatology subgroup) (Figure 2B).

As expected, patients first seen by a pulmonologist more frequently had ILD as compared to those first evaluated by a rheumatologist [48 (92.3%) vs. 27 (30.7%), *p* < 0.001); they also more frequently had isolated ILD [22 (42.3%) vs. 10 (11.4%), *p* < 0.001]. Muscle weakness [15 (30.8%) vs. 53 (60.2%), *p* < 0.001] and skin manifestations [27 (51.9%) vs. 61 (69.3%), *p* = 0.04] were significantly less frequently present in the “pulmonology first” subgroup of patients, as opposed to dyspnea, which was more frequently reported in this subgroup [33 (80.5%) vs. 9 (10.8%), *p* < 0.001] (Figure 2C).

Regarding laboratory findings, CK [median 103.5 IU/L (IQR 48.5–676.5) vs. 301.5 IU/L (81.3–1356.0), *p* = 0.013] and AST [22 IU/L (16.0–37.0) vs. 30.5 IU/L (19.3–61.8), *p* = 0.011] were lower in patients first evaluated by a pulmonologist (Table 2, Figure 2D).

Anti-tRNA synthetase antibodies were more frequently detected in the pulmonology subgroup (Figure 2E). Demographic, clinical and serological characteristics according to the first evaluating medical specialty are shown in Table 2.

## 4. Discussion

In this contemporary IIM cohort, we found that patients with ILD presented with distinct clinical characteristics compared to those without ILD. This phenotypic divergence appears to affect early care pathways, determining whether patients initially present to pulmonology versus rheumatology services.

From a clinical perspective, although our overall cohort was predominantly female, with an approximate female-to-male ratio of 2:1, patients with IIM-ILD were significantly more likely to be male (OR = 2.43) and older compared to patients with no ILD, with a mean age of approximately 60 years. An older age at diagnosis of IIM is an established risk factor of ILD occurrence [12,13]. Notably, this demographic profile overlaps with the typical clinical phenotype of idiopathic pulmonary fibrosis (IPF), namely males over 60 years of age [11,14]. Accordingly, these findings highlight the risk of a “reversed Yentl syndrome” [15] in IIM-associated ILD, whereby male sex and older age may steer clinical reasoning away from an autoimmune etiology, towards a diagnosis of IPF, potentially delaying early diagnosis and timely management of IIM-ILD.

As expected, we found that dyspnea was more prevalent among patients with IIM-ILD and in those who initially presented to a pulmonology service. In fact, only one of 65 patients with IIM without ILD reported dyspnea. While this may seem self-evident, it has important clinical implications. Specifically, although dyspnea in IIM can result from respiratory muscle involvement [2], our cohort suggests that this is uncommon in everyday practice. Instead, the presence of dyspnea most often reflects underlying lung involvement.

Clinical muscle involvement, manifesting as muscle weakness, was less common in IIM patients with ILD and in those initially evaluated by a pulmonologist. This is also reflected in the fact that CK, SGOT and LDH levels were lower in patients with ILD and that CK and SGOT were lower in patients first evaluated by a pulmonologist. These findings are consistent with those of a study by Barratt et al. [16], in which patients with ASyS presenting initially to pulmonologists less frequently reported muscle weakness or demonstrated biochemical markers of myositis than those first seen by rheumatologists. Similarly, Huang et al. [17] reported that patients with IIM and ILD had lower CK levels compared with patients without ILD, while Cheng et al. [18] showed that lower CK levels were associated with the occurrence of ILD in IIM patients. Accordingly, in our study, patients first presenting to a pulmonologist were more likely to have isolated ILD. This is a common clinical scenario as ILD can be the first or sole manifestation in the setting of IIM, especially ASyS [1]. In a large multicentric study of patients with ASyS, isolated ILD at presentation was the most common manifestation, being present in approximately 18.5% of 828 patients [19]. Overall, 16–65% of IIM patients present with isolated ILD, depending on the study and the specific serological profile [2].

CRP levels were significantly higher in patients with IIM-associated ILD. This observation is consistent with a retrospective study of 130 patients with IIM, including 55 with IIM-ILD, in which elevated CRP levels were positively associated with an increased risk of developing ILD [13]. In another study, CRP levels were also higher in patients with IIM-ILD compared with those without ILD; however, CRP was not identified as an independent risk factor for ILD development in IIM [20]. In a meta-analysis, elevated CRP levels were associated with the development of ILD in patients with IIM [12]. This finding reflects not only the increased inflammatory burden in patients with IIM-ILD but also, from a pathogenetic viewpoint, the potential role of the lung as the initiating site of inflammation and the primary target of the autoimmune response [21]. Interestingly, respiratory tract infections have been associated with an increased risk of IIM [22,23], and this effect seems to be more pronounced among patients with myositis and concurrent interstitial lung disease [24]. In addition to infectious triggers, environmental factors may contribute substantially to the development of IIM by inducing persistent immune activation in individuals with a genetic predisposition. Up to 50–59% of patients with IIM report occupational, domestic, or environmental exposures [25,26], while in those with predominant lung involvement, 37–43% have a history of contact with birds or feather-filled bedding [25,27].

In addition to differences in laboratory findings, distinct serological profiles were observed, with patients with ILD and those initially evaluated by a pulmonologist more likely to test positive for anti-tRNA synthetase and anti-Ro52 antibodies. ILD is an extremely common manifestation of ASyS affecting more than 80% of patients [1,2], while Ro52 positivity has consistently been associated with increased frequency and severity of ILD [28,29].

According to our results, patients with IIM-ILD exhibit distinct clinical characteristics compared with those without ILD, resulting in a unique clinical phenotype. Specifically, they are more likely to present without overt clinical or biochemical manifestations of myositis, rendering isolated ILD the predominant presentation. In our cohort, isolated ILD was observed in 22.9% of patients. Similarly, in a series from the Mayo Clinic including 70 patients with polymyositis–dermatomyositis (PM–DM)-associated ILD, approximately 30% did not exhibit overt extrapulmonary manifestations of PM–DM [30].

This divergence strongly influences the initial evaluating specialty. Specifically, pulmonologists and rheumatologists may encounter patients with the same underlying disease but markedly different phenotypic expressions. Pulmonologists, as expected, tend to prioritize diagnostic entities that are more familiar and commonly encountered in respiratory practice, making the diagnosis of IIM-ILD challenging [31]. In the presence of relevant environmental exposures, hypersensitivity pneumonitis is often considered [32], whereas in older male patients, idiopathic pulmonary fibrosis is more frequently favored [11,33]. Importantly, ILD is not included in the current EULAR/ACR classification criteria for IIM [9], which place greater emphasis on muscle involvement, laboratory abnormalities, and histopathology. However, accumulating evidence demonstrates that these criteria may fail to capture cases in which the lung represents the sole or predominant organ of involvement. This limitation can have important clinical implications, as ILD is a major driver of morbidity and mortality in IIM [1].

Characteristic cutaneous findings are highly informative and, in some cases, pathognomonic in IIM; however, in our study, no differences were observed in skin manifestations or Raynaud’s phenomenon between IIM patients with and without ILD. Similarly, in a retrospective study of 135 patients with IIM, including 55 with IIM-ILD, there were no differences in the prevalence of mechanic’s hands or Raynaud’s phenomenon between the groups [13]. Consistently with this, a study of 35 patients with clinically amyopathic ILD associated with myositis-specific autoantibodies, including 28 patients with ASyS, reported mechanic’s hands in 31% of cases, Gottron papules in 8%, and Raynaud’s phenomenon in 23% [27].

This observation can help refine our understanding of clinically amyopathic dermatomyositis (CADM) in the modern era. The term CADM was coined in 1993 to describe patients with cutaneous manifestations of classic dermatomyositis but lacking objective evidence of myositis [34]. Notably, patients with CADM are more likely to have arthritis and ILD [35]. At that time, myositis autoantibody panels were not available, and clinicians therefore relied primarily on characteristic cutaneous findings to suspect a diagnosis within the spectrum of IIM. Moreover, HRCT was not part of routine clinical practice, limiting the early and reliable detection of ILD. In contrast, in the contemporary era, the widespread availability and high specificity of myositis autoantibody panels have shifted diagnostic paradigms, with serological testing increasingly complementing clinical examination. The key clinical message is that amyopathic presentations are not confined to patients with cutaneous involvement; clinically amyopathic myositis may occur in the absence of skin manifestations, and a substantial proportion of these patients develop interstitial lung disease, which has a profound impact on prognosis and survival.

The clinical differences observed between patients with and without ILD should be interpreted within the context of their underlying serological profile. In our cohort, patients with ILD were enriched for anti-tRNA synthetase antibodies, reflecting a higher prevalence of ASyS within this subgroup. This serological predominance likely contributes substantially to the observed phenotype, including the higher frequency of pulmonary manifestations, lower muscle enzyme levels, reduced prevalence of overt muscle weakness, and greater likelihood of initial evaluation by pulmonologists. Therefore, the differences identified in our analysis should not be interpreted as effects of ILD status alone. Rather, they probably reflect the close relationship between ILD and specific immunological subsets of IIM, particularly ASyS, in which lung involvement often represents the dominant clinical manifestation. Consequently, the ILD status and serological profile should be viewed as interrelated rather than independent determinants of clinical presentation.

Our findings provide real-world validation of previously described phenotypic patterns in IIM, particularly the subset of patients presenting with predominant or isolated ILD in the absence of clinically evident myositis. The observed differences in clinical presentation, laboratory profiles, and referral pathways are consistent with prior reports. Recognizing these differences is important, as it may influence diagnostic thinking, referral decisions, and the timely initiation of appropriate therapy.

## 5. Conclusions

Our study has several limitations. The cohort size was relatively modest, though adequate given the rarity of the disease. Certain myositis subtypes were underrepresented, which may limit the applicability of our findings across the full spectrum of IIM. Also, radiological imaging data were not available for analysis. The initial evaluation specialty is influenced by the presenting phenotype and may introduce circularity; however, its inclusion provides insight into real-world diagnostic pathways rather than causal relationships. A further limitation is that the ILD subgroup was enriched for anti-tRNA synthetase antibodies and ASyS, making it difficult to fully disentangle the effects of ILD status from those of the underlying serological subset. In addition, as this was a single-center study, the generalizability of the results may be restricted. Finally, the study has the inherent limitations of a retrospective design.

In conclusion, patients with IIM-ILD, particularly those belonging to ASyS-associated serological subsets, often present with a distinct clinical phenotype in which the lung is the predominant or sole involved organ. This observation underscores the need for increased recognition of this clinical phenotype by both pulmonology and rheumatology specialists and highlights the importance of close interdisciplinary collaboration. It is important that future classification criteria for IIM incorporate the lung as a predominant organ of disease involvement. In this context, comprehensive testing for myositis-specific autoantibodies is particularly valuable, as the detection of characteristic autoantibodies may support the diagnosis without the need for additional invasive diagnostic procedures. This strategy supports the implementation of personalized medicine by enabling timely and accurate diagnosis, facilitating earlier initiation of appropriate therapy and ultimately improving patient outcomes [1,36].

## Figures and Tables

**Table 1:** Demographics and clinical characteristics (n = 140).

Characteristic	Total Cohort	Without ILD	With ILD	*p*-Value
	140	65 (46.4)	75 (53.6)	
Age, mean (SD), years	55.8 (15.7)	50.7 (17.4)	60.2 (12.5)	<0.001
Sex, male, n (%)	44 (31.4)	14 (21.5)	30 (40.0)	0.019
Sex, female, n (%)	96 (68.6)	51 (78.5)	45 (60.0)	
Smoking status, n (%)	n = 119	n = 49	n = 70	0.001
Current	20 (16.8)	15 (30.6)	5 (7.1)	
Ex-smoker	30 (25.2)	7 (14.3)	23 (32.9)	
Never	69 (58.0)	27 (55.1)	42 (60.0)	
Symptoms, n (%)				
Muscle weakness	69 (49.3)	47 (72.3)	22 (29.3)	<0.001
Skin manifestations	88 (62.9)	41 (63.1)	47 (62.7)	0.960
Dyspnea (n = 124)	42 (30.0)	1 (1.7)	41 (64.1)	<0.001
Arthritis	74 (52.9)	35 (53.8)	39 (52.0)	0.827
Raynaud	28 (20.0)	11 (16.9)	17 (22.7)	0.397
Laboratory, median (IQR)				
CK levels (IU/L)	171 (68.0–1057.3)	580 (107.5–2674.5)	103 (50.8–331.8)	<0.001
SGOT (IU/L)	26 (18–50.5)	38 (20.5–95.0)	22 (16–36)	<0.001
SGPT (IU/L)	28 (18.0–55.0)	34 (22.0–82.5)	26 (17.5–47)	0.033
LDH (IU/L)	275 (211.5–435.5)	315 (227.0–467.3)	244 (190.0–410.0)	0.009
ESR (mm)	20.0 (10.0–40.0)	20.0 (8.3–30.0)	22.0 (11.5–44.5)	0.158
CRP (mg/L)	3.9 (1.4–11.0)	2.9 (1.0–8.0)	5.95 (2.0–14.1)	0.024
Autoantibody, n (%)				
Anti-tRNA	42 (30.0)	8 (12.3)	34 (45.3)	<0.001
Anti-MDA5	15 (10.7)	7 (10.8)	8 (10.7)	0.984
Anti-SRP	10 (7.1)	6 (9.2)	4 (5.3)	0.514
Anti-SAE1	4 (2.9)	3 (4.6)	1 (1.3)	0.337
Anti-Mi2	11 (7.9)	5 (7.7)	6 (8.0)	0.946
Anti-NXP-2	9 (6.4)	6 (9.2)	3 (4.0)	0.303
Anti-Tiff-1gamma	7 (5.0)	6 (9.2)	1 (1.3)	0.049
Anti-HMGCR	4 (2.9)	3 (4.6)	1 (1.3)	0.337
Anti-cN1a	8 (5.7)	8 (12.3)	0 (0.0)	0.002
Anti-Ro52	43 (30.7)	5 (7.7)	38 (50.7)	<0.001
Anti-PM/Scl	14 (10.0)	3 (4.6)	11 (14.7)	0.048
Anti-Ku	7 (5.0)	2 (3.1)	5 (6.7)	0.450

Percentages were calculated within each subgroup.

**Table 2:** Baseline characteristics of patients according to initial evaluation specialty (pulmonology vs. rheumatology).

Characteristic	Total Cohort (n = 140)	Initial Visit to Rheumatologist (n = 88)	Initial Visit to Pulmonologist (n = 52)	*p*-Value
Age, mean (SD), years	55.8 (15.7)	53 (17.3)	60.5 (11.2)	0.002
Sex, male, n (%)	44 (31.4)	23 (26.1)	21 (40.4)	0.079
Sex, female, n (%)	96 (68.6)	65 (73.9)	31 (59.6)	
Smoking status, n (%)	n = 119	n = 70	n = 49	0.047
Current	20 (16.8)	14 (20.0)	6 (12.2)	
Ex-smoker	30 (21.4)	12 (17.1)	18 (36.7)	
Never	69 (58.0)	44 (62.9)	25 (51.0)	
ILD, n (%)	75 (53.6)	27 (30.7)	48 (92.3)	<0.001
Isolated lung involvement, n (%)	32 (22.9)	10 (11.4)	22 (42.4)	<0.001
Symptoms, n (%)				
Muscle weakness	69 (49.3)	53 (60.2)	16 (30.8)	<0.001
Skin manifestations	88 (62.9)	61 69.3)	27 (51.9)	0.040
Dyspnea (n = 124)	42 (30.0)	9 (10.8)	33 (80.5)	<0.001
Arthritis	74 (52.9)	45 (51.1)	29 (55.8)	0.596
Raynaud	28 (20.0)	19 (21.6)	9 (17.3)	0.540
Laboratory, median (IQR)				
CK levels (IU/L)	171.0 (68.0–1057.3)	301.5 (81.3–1356.0)	103.5 (48.5–676.5)	0.013
SGOT (IU/L)	26.0 (18–50.5)	30.5 (19.3–61.8)	22.0 (16.0–37.0)	0.011
SGPT (IU/L)	28.0 (18.0–55.0)	29.0 (18.0–71.0)	26.0 (18–46.5)	0.309
LDH (IU/L)	275.0 (211.5–435.5)	285.0 (220.0–458.0)	256.0 (191.5–433.0)	0.212
ESR (mm)	20.0 (10.0–40.0)	20.5 (10.0–44.3)	20.0 (10.0–35.0)	0.900
CRP (mg/L)	3.9 (1.4–11.0)	3.6 (1.1–11.2)	5.7 (1.7–10.9)	0.230
Autoantibody, n/N (%)				
Anti-tRNA	42 (30.0)	15 (17.2)	24 (50.0)	<0.001
Anti-MDA5	15 (10.7)	11 (12.6)	4 (8.3)	0.446
Anti-SRP	10 (7.1)	6 (6.9)	3 (6.3)	1.000
Anti-SAE	4 (2.9)	3 (3.4)	1 (2.1)	1.000
Anti-Mi2	11 (7.9)	5 (5.7)	6 (12.5)	0.198
Anti-NXP-2	9 (6.4)	6 (6.9)	2 (4.2)	0.711
Anti-Tiff-1gamma	7 (5.0)	7 (8.2)	0 (0.0)	0.050
Anti-HMGCR	4 (2.9)	4 (4.6)	0 (0.0)	0.297
Anti-cN1a	8 (5.7)	6 (6.9)	2 (4.2)	0.711
Anti-Ro52	43 (30.7)	21 (24.1)	19 (39.6)	0.060
Anti-PM/Scl	14 (10.0)	6 (6.9)	8 (16.7)	0.085
Anti-Ku	7 (5.0)	4 (4.6)	3 (6.3)	0.699

Percentages were calculated within each subgroup.

## Data Availability

The data that support the findings of this study are available from the corresponding author upon reasonable request.

## Supplementary Materials

The following supporting information can be downloaded at https://www.mdpi.com/article/10.3390/jpm16060311/s1, Figure S1. Cumulative characteristics of the cohort of patients with IIM. Bar and Box plots depict (A) sex distribution, (B) smoking status, (C) symptoms and (D) laboratory findings and (E) auto-antibody profile.

## Abbreviations

IIM	Idiopathic inflammatory myopathy
ILD	Interstitial lung disease
PM	Polymyositis
DM	Dermatomyositis
AsyS	Antisynthetase syndrome
CADM	Clinically amyopathic dermatomyositis
IPF	Idiopathic pulmonary fibrosis
HRCT	High-Resolution Computed Tomography
CPK	Creatine kinase
LDH	Lactate Dehydrogenase
CRP	C-reactive Protein
SGOT	Serum Glutamic–Oxaloacetic Transaminase
SGPT	Serum Glutamic–Pyruvic Transaminase
SD	Standard deviation
IQR	Interquartile range
ln	Natural logarithm

## References

[1] Hallowell R.W., Danoff S.K. (2023). Diagnosis and Management of Myositis-Associated Lung Disease. Chest.

[2] Sehgal S., Patel A., Chatterjee S., Fernandez A.P., Farver C., Yadav R., Li Y., Danoff S.K., Saygin D., Huapaya J.A. (2025). Idiopathic inflammatory myopathies related lung disease in adults. Lancet Respir. Med..

[3] Johnson C., Pinal-Fernandez I., Parikh R., Paik J., Albayda J., Mammen A.L., Christopher-Stine L., Danoff S. (2016). Assessment of Mortality in Autoimmune Myositis with and Without Associated Interstitial Lung Disease. Lung.

[4] Aggarwal R., Cassidy E., Fertig N., Koontz D.C., Lucas M., Ascherman D.P., Oddis C.V. (2014). Patients with non-Jo-1 anti-tRNA-synthetase autoantibodies have worse survival than Jo-1 positive patients. Ann. Rheum. Dis..

[5] Kiely P.D., Chua F. (2013). Interstitial lung disease in inflammatory myopathies: Clinical phenotypes and prognosis. Curr. Rheumatol. Rep..

[6] Li Y., Gao X., Li Y., Jia X., Zhang X., Xu Y., Gan Y., Li S., Chen R., He J. (2020). Predictors and Mortality of Rapidly Progressive Interstitial Lung Disease in Patients with Idiopathic Inflammatory Myopathy: A Series of 474 Patients. Front. Med..

[7] Shappley C., Paik J.J., Saketkoo L.A. (2019). Myositis-Related Interstitial Lung Diseases: Diagnostic Features, Treatment, and Complications. Curr. Treat. Options Rheumatol..

[8] Jiang W., Shi J., Yang H., Tian X., Yang H., Chen Q., Zhang L., Peng Q., Wang G., Lu X. (2023). Long-Term Outcomes and Prognosis Factors in Patients with Idiopathic Inflammatory Myopathies Based on Myositis-Specific Autoantibodies: A Single Cohort Study. Arthritis Care Res..

[9] Lundberg I.E., Tjärnlund A., Bottai M., Werth V.P., Pilkington C., Visser M., Alfredsson L., Amato A.A., Barohn R.J., Liang M.H. (2017). 2017 European League Against Rheumatism/American College of Rheumatology classification criteria for adult and juvenile idiopathic inflammatory myopathies and their major subgroups. Ann. Rheum. Dis..

[10] Connors G.R., Christopher-Stine L., Oddis C.V., Danoff S.K. (2010). Interstitial lung disease associated with the idiopathic inflammatory myopathies: What progress has been made in the past 35 years?. Chest.

[11] Raghu G., Remy-Jardin M., Richeldi L., Thomson C.C., Inoue Y., Johkoh T., Kreuter M., Lynch D.A., Maher T.M., Martinez F.J. (2022). Idiopathic Pulmonary Fibrosis (an Update) and Progressive Pulmonary Fibrosis in Adults: An Official ATS/ERS/JRS/ALAT Clinical Practice Guideline. Am. J. Respir. Crit. Care Med..

[12] Zhang L., Wu G., Gao D., Liu G., Pan L., Ni L., Li Z., Wang Q. (2016). Factors Associated with Interstitial Lung Disease in Patients with Polymyositis and Dermatomyositis: A Systematic Review and Meta-Analysis. PLoS ONE.

[13] Wang Z., Zhang J., Li J., Mao X., Li Y., Wang D., Ge W., Li J., Liang C., Zhang Z. (2025). Integration of clinical and serological biomarkers in a nomogram for predicting interstitial lung disease in idiopathic inflammatory myopathies. BMC Rheumatol..

[14] Tzilas V., Tzouvelekis A., Chrysikos S., Papiris S., Bouros D. (2017). Diagnosis of Idiopathic Pulmonary Fibrosis “Pragmatic Challenges in Clinical Practice”. Front. Med..

[15] Healy B. (1991). The Yentl syndrome. N. Engl. J. Med..

[16] Barratt S.L., Adamali H.H., Cotton C., Mulhearn B., Iftikhar H., Pauling J.D., Spencer L., Adamali H.I., Gunawardena H. (2021). Clinicoserological features of antisynthetase syndrome (ASyS)-associated interstitial lung disease presenting to respiratory services: Comparison with idiopathic pulmonary fibrosis and ASyS diagnosed in rheumatology services. BMJ Open Respir. Res..

[17] Huang H.L., Lin W.C., Yeh C.C., Sun Y.T. (2020). Serological risk factors for concomitant interstitial lung disease in patients with idiopathic inflammatory myopathy. J. Clin. Neurosci..

[18] Cheng L., Li Y., Luo Y., Zhou Y., Wen J., Wu Y., Liang X., Wu T., Tan C., Liu Y. (2023). Decreased Th1 Cells and Increased Th2 Cells in Peripheral Blood Are Associated with Idiopathic Inflammatory Myopathies Patients with Interstitial Lung Disease. Inflammation.

[19] Cavagna L., Trallero-Araguás E., Meloni F., Cavazzana I., Rojas-Serrano J., Feist E., Zanframundo G., Morandi V., Meyer A., Pereira da Silva J.A. (2019). Influence of Antisynthetase Antibodies Specificities on Antisynthetase Syndrome Clinical Spectrum Time Course. J. Clin. Med..

[20] Cen X., Zuo C., Yang M., Yin G., Xie Q. (2013). A clinical analysis of risk factors for interstitial lung disease in patients with idiopathic inflammatory myopathy. Clin. Dev. Immunol..

[21] Danoff S.K., Casciola-Rosen L. (2011). The lung as a possible target for the immune reaction in myositis. Arthritis Res. Ther..

[22] Svensson J., Holmqvist M., Lundberg I.E., Arkema E.V. (2017). Infections and respiratory tract disease as risk factors for idiopathic inflammatory myopathies: A population-based case-control study. Ann. Rheum. Dis..

[23] Ohnishi T., Wilkerson J., Bayat N., Farhadi P.N., Faiq A., Dillon C.F., Schiffenbauer A., Parks C.G., Brunner H.I., Goldberg B. (2025). Infections preceding diagnosis associated with myositis phenotypes in a national patient registry. Clin. Exp. Rheumatol..

[24] Helmers S.B., Jiang X., Pettersson D., Wikman A.L., Axelman P., Lundberg Å., Lundberg I.E., Alfredsson L. (2016). Inflammatory lung disease a potential risk factor for onset of idiopathic inflammatory myopathies: Results from a pilot study. RMD Open.

[25] Costa A.N., Kawano-Dourado L., Shinjo S.K., Carvalho C.R., Kairalla R.A. (2014). Environmental exposure in inflammatory myositis. Clin. Rheumatol..

[26] Labirua-Iturburu A., Selva-O’Callaghan A., Zock J.P., Orriols R., Martínez-Gómez X., Vilardell-Tarrés M. (2014). Occupational exposure in patients with the antisynthetase syndrome. Clin. Rheumatol..

[27] Tzilas V., Tzouvelekis A., Sotiropoulou V., Panopoulos S., Bouros E., Avdoula E., Ryu J.H., Bouros D. (2024). Presenting clinical and imaging features of patients with clinically amyopathic interstitial lung disease associated with myositis-specific autoantibodies. Front. Med..

[28] Gui X., Shenyun S., Ding H., Wang R., Tong J., Yu M., Zhao T., Ma M., Ding J., Xin X. (2022). Anti-Ro52 antibodies are associated with the prognosis of adult idiopathic inflammatory myopathy-associated interstitial lung disease. Rheumatology.

[29] Nayebirad S., Mohamadi A., Yousefi-Koma H., Javadi M., Farahmand K., Atef-Yekta R., Tamartash Z., Jameie M., Mohammadzadegan A.M., Kavosi H. (2023). Association of anti-Ro52 autoantibody with interstitial lung disease in autoimmune diseases: A systematic review and meta-analysis. BMJ Open Respir. Res..

[30] Douglas W.W., Tazelaar H.D., Hartman T.E., Hartman R.P., Decker P.A., Schroeder D.R., Ryu J.H. (2001). Polymyositis-dermatomyositis-associated interstitial lung disease. Am. J. Respir. Crit. Care Med..

[31] Tzilas V., Ryu J.H., Sfikakis P.P., Tzouvelekis A., Bouros D. (2023). Antisynthetase syndrome with predominant lung involvement. An easy to miss diagnosis. Pulmonology.

[32] Tzilas V., Sfikakis P.P., Bouros D. (2021). Antisynthetase Syndrome Masquerading as Hypersensitivity Pneumonitis. Respiration.

[33] Tzilas V., Tzouvelekis A., Ryu J.H., Bouros D. (2022). 2022 update on clinical practice guidelines for idiopathic pulmonary fibrosis and progressive pulmonary fibrosis. Lancet Respir. Med..

[34] Euwer R.L., Sontheimer R.D. (1993). Amyopathic dermatomyositis: A review. J. Investig. Dermatol..

[35] Fornaro M., Girolamo F., Giannini M., Coladonato L., Capuano A., Capodiferro M., D’Abbicco D., Ruggieri M., Mastrapasqua M., Iannone F. (2024). Clinical, histologic and prognostic features of clinically amyopathic dermatomyositis. Clin. Exp. Rheumatol..

[36] Alrehaili G., Farah W., Tzilas V., Baqir M. (2025). Idiopathic Inflammatory Myositis (IIM) Related Interstitial Lung Disease (ILD): Treatment Options. Curr. Pulmonol. Rep..

