# Supplementary figures and images for: Clinical and Serological Characteristics of Idiopathic Inflammatory Myopathies According to the Presence of Interstitial Lung Disease and Initial Evaluating Medical Specialty: A Single-Center Experience
Source: J Pers Med. 2026 Jun 8;16(6):311. doi: 10.3390/jpm16060311 (PMC13300778; doi:10.3390/jpm16060311)

# Supplementary Figure S1

A.

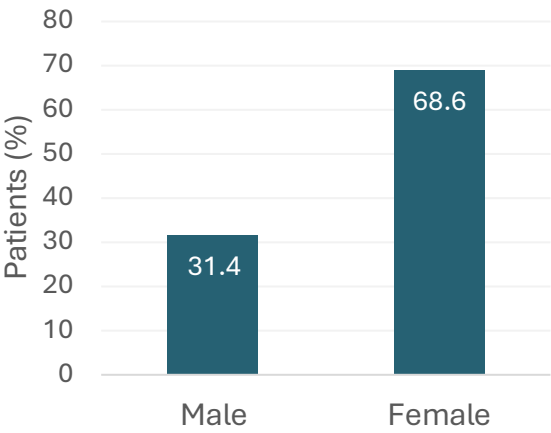

B.

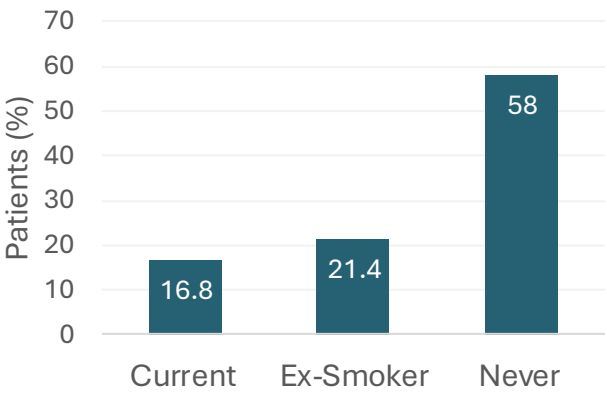

C.

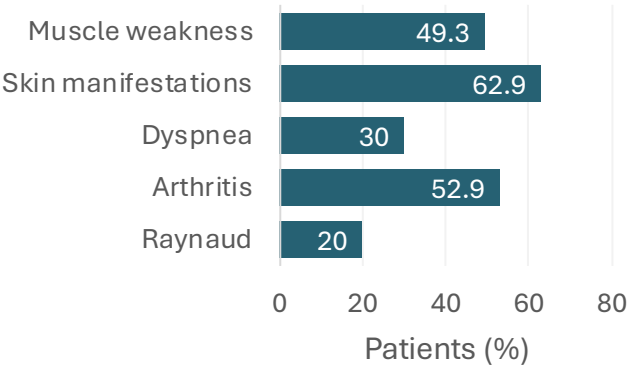

D.

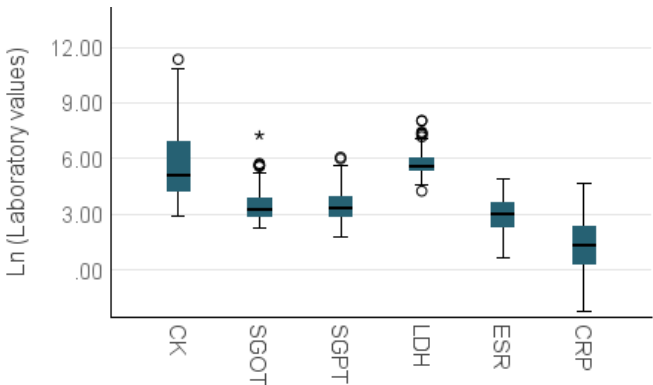

E.

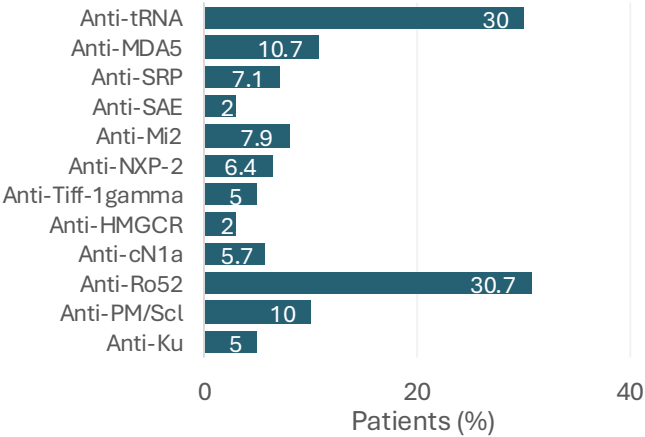

Supplement: Supplementary file 1 [file jpm-16-00311-s001.zip › jpm-4246233-supplementary.pdf]
